# Association between albuminuria and thyroid function in patients with chronic kidney disease

**DOI:** 10.1007/s12020-021-02640-1

**Published:** 2021-02-11

**Authors:** Walter Reinhardt, Nils Mülling, Stefan Behrendt, Sven Benson, Sebastian Dolff, Dagmar Führer, Susanne Tan

**Affiliations:** 1 Department of Nephrology, University Hospital Essen, University Duisburg-Essen, Hufelandstr. 55, 45147 Essen, Germany; 2 Institute of Medical Psychology and Behavioral Immunobiology, University Hospital Essen, University Duisburg-Essen, Hufelandstr. 55, 45147 Essen, Germany; 3 Department of Infectious Diseases, University Hospital Essen, University Duisburg-Essen, Hufelandstr. 55, 45147 Essen, Germany; 4 Department of Endocrinology and Metabolism, Division of Laboratory Research, University Hospital Essen, University Duisburg-Essen, Hufelandstr. 55, 45147 Essen, Germany

**Keywords:** Chronic kidney disease, Kidney function, Kidney damage, Albuminuria, Thyroid function, Reverse triiodothyronine

## Abstract

**Purpose:**

The relationship between proteinuria and thyroid function remains controversial in patients with chronic kidney disease (CKD). We prospectively investigated the association between kidney and thyroid function in thyroid antibody-negative patients through all CKD stages.

**Methods:**

We enrolled 184 nondialysis patients (mean age: 63.1 ± 16.9 years) without previous thyroid disease or thyroid-specific antibodies. Kidney function was assessed by estimating the glomerular filtration rate (eGFR) classified according KDIGO (CKD G1–5). Kidney damage was assessed by albuminuria (albumin-to-creatinine ratio, ACR) and classified as mild, moderate, or severe (ACR1: <300, ACR2: 300–3000, and ACR3: 3000 mg/g). To evaluate thyroid function, TSH, T4, fT4, T3, fT3, reverse T3 (rT3), and thyroxine-binding globulin (TBG) were measured.

**Results:**

rT3 concentrations correlated negatively with albuminuria (*r* = −0.286, *p* < 0.001) and were significantly lower in patients with severe albuminuria than in those with mild or moderate albuminuria (ACR3: 0.28 vs. ACR2: 0.32 vs. ACR1: 0.36 nmol/l, *p* < 0.001). The severity of albuminuria revealed no impact on TSH, fT4, T3, fT3, and TBG. EGFR correlated with increasing T4, fT4, T3, fT3, and TBG (T4: *r* = 0.289, *p* < 0.01; fT4: *r* = 0.196, *p* < 0.01; T3: *r* = 0.408, *p* < 0.01; fT3: *r* = 0.390, *p* < 0.01) but not with rT3.

**Conclusions:**

In thyroid antibody-negative patients presenting advanced CKD (stages 4 and 5), even severe kidney protein loss failed to influence thyroid hormone status. However, albuminuria severity correlated negatively with rT3, which was significantly lower in patients with albuminuria in the nephrotic range.

## Introduction

Proteinuria is common in patients with chronic kidney disease (CKD), e.g., the glomerulonephritis and renal complications caused by diabetes mellitus, and has become a prognostic variable in CKD patients regarding mortality, cardiovascular events, and progression to end-stage kidney disease [1, 2]. However, the relationship between proteinuria in CKD and thyroid function remains unclear. It is well known that thyroid hormones in serum are bound to thyroxine-binding globulin (TBG), albumin, and transthyretin. Thus, proteinuria in the nephrotic range (>3 g/day) is associated with the loss of TBG, levothyroxine (T4), or both, leading to (subclinical) hypothyroidism, especially in children [3–10]. Moreover, the dose of thyroid replacement therapy must be increased in patients with hypothyroidism and concomitant nephrotic syndrome [11–13].

Thyroid function is also strongly influenced by kidney function. Nonthyroidal illness (NTI) or euthyroid sick syndrome is very common among ill patients [14]. Also, CKD patients demonstrate low thyroid hormone parameters, but in contrast to other forms of NTI, reverse T3 (rT3) levels are normal or even low [9, 10, 15–19]. rT3 itself exhibits no thyromimetic activity.

The severity of NTI also reflects the prognosis for hemodialysis and patients after renal transplantation (RTx) [20–22].

There have only been case reports to date on this issue, and the numbers of investigated patients have been low. Four studies assessed thyroid function in larger patient cohorts with proteinuria and CKD, but they arrived at different results

These studies were mainly carried out retrospectively in patients with good or slightly impaired renal function [23–26]. Only a Korean working group also included patients with CKD stages 3 and 4 [27]. Moreover, in several studies thyroid antibody status was not reported [24, 26, 27].

Therefore, we prospectively investigated the association of proteinuria and renal function with thyroid hormone status in thyroid antibody-negative patients.

## Patients and methods

### Study design and participants

Inclusion and exclusion criteria: Patients examined in our outpatient clinic for further work-up of presumed kidney damage were evaluated prospectively as previously published to examine the link between selenoprotein P, thyroid hormone, and renal impairment [28]. Thus, serum samples were drawn from July 2013 to July 2014. After centrifugation, serum samples were stored frozen (−80°) and analyzed between August 2014 and October 2014.

Reasons for study exclusion were previous known thyroid disorder requiring thionamides or levothyroxine treatment, and/or presence of antibodies against thyroid peroxidase, as well as medication with known or potential influence on thyroid hormone function (current or past intake of amiodarone, a high-dose steroid or furosemide, anticonvulsives, heparin, or estrogen-replacement therapy). Our local ethics committee approved the study. All patients gave informed consent to undergo testing and to be included in this pseudonymized analysis.

### Laboratory diagnostics

To assess kidney function, we calculated the estimated glomerular filtration rate (eGFR) by referring to the abbreviated modification of diet in renal disease formula [29]. CKD stages were defined by eGFR according to the KDIGO: stage 1–2: >60; stage 3a: 45–59, stage 3b: 30–44; stages 4–5 < 30 ml/min/1.73 m^2^. Serum and urinary albumin and creatinine and serum c-reactive protein (CRP) were determined by standard methods. Albuminuria was assessed by albumin-to-creatinine ratio (ACR) and classified as mild, moderate, or severe when ACR < 300 (ACR1), 300–3000 (ACR2), and >3000 mg/g (ACR3). Automated chemiluminescence immunoassay systems (ADVIA Centaur, Bayer Vital, Fernwald, Germany) were used to determine thyrotropin (TSH), T4, free T4 (fT4), triiodothyronine (T3), fT3, and TBG concentrations in serum. rT3 was measured by RIA (Fa. Adaltis, Freiburg, Germany; with rT3 antiserum raised in rabbit; normal range: 0.14–0.54 nmol/l; sensitivity range: 0.03–3.1 nmol/l, % cross reactivity: T4: 0.029; T3: 0.0086). Anti-TPO Abs were measured by the Immulite 2000 Anti-TPO Ab assay; a solid-phase enzyme-labeled chemiluminescent sequential immunometric assay. The cut off level < 35 U/ml was provided by the manufacturer.

All laboratory testing was done at the University Hospital Essen Division of Laboratory Research by technicians blinded to patients’ clinical data.

### Statistical analysis

Correlations were calculated as Spearman rho. In addition, partial correlation analyses were completed to control for age and CRP. Thyroid and renal parameters were compared with Kruskal–Wallis tests, followed by Bonferroni-corrected Mann–Whitney *U* tests controlling for multiple testing. Our results are presented as median [25th–75th percentiles] or mean ± standard deviation, as indicated. The alpha level for significance was 0.05.

## Results

### Demographics

A total of 294 patients with underlying CKD were screened. Of those patients, the following had to be excluded: 15 were TPO Ab positive, 32 were taking thyroid hormones (due to autoimmune thyroid disease or after thyroid surgery), 5 thionamides, 40 were taking prednisolone > 5 mg/day, 4 amiodarone, 4 anticonvulsives, and 10 estrogens.

The nature of kidney disease: 27% diabetes type 2, 3% diabetes type 1, 28% nephrosclerosis, 19% glomerulonephritis, 8% polycystic kidney disease, 10% interstitial nephritis, and 5% unknown.

Thus, 184 patients were enrolled in the study. A total of 53 patients were taking oral antiglycemic medication and/or insulin, 130 antihypertensive medication, and 30 had elevated lipids and were on statins. None of the patients were on a restricted diet. Blood samples were taken between 8 and 11 a.m. in the morning after an overnight fast.

No or mild (G1–2), moderate (G3), and severe (G4–5) impaired kidney function was present in 33 (18%), 68 (37%), and 83 (45%) patients, respectively. Albuminuria was mild, moderate, and severe in 80 (43%), 83 (45%), and 21 (12%) cases, respectively. ACR was not associated with eGFR (*r* = 0.051; *p* = 0.483). Only 1.6% revealed TSH levels > 4 mU/l (range 4.4–13.6) and 3.3% had TSH levels < 0.3 mU/l.

Patients’ characteristics are provided in detail in Table 1.

**Table 1:** Patient characteristics (*n* = 184)

Variable	Value
Age (years)	63.1 ± 16.9
BMI (kg/m^2^)	29.4 ± 5.6
CRP (mg/dl; normal range: <0.5 mg/l)	0.4 (0.2–0.8)
eGFR (ml/min/1.73 m^2^)	25.73 (16.36–45.12)
CKD G1–2 (*n*(%))	33 (18%)
CKD G3a–b (*n*(%))	68 (37%)
CKD G4–5 (*n*(%))	83 (45%)
Creatinine (mg/dl; normal range: 0.67–1.17 mg/dl)	2.01 (1.40–2.95)
Serum albumin (mg/dl; normal range: 3.5–5.5 mg/dl)	4.2 (3.9–4.4)
Urinary albumin (mg/g creatinine; normal range: <30 mg/g creatinine)	469.5 (61.5–1748.0)
ACR1 (*n*(%))	80 (43%)
ACR2 (*n*(%))	83 (45%)
ACR3 (*n*(%))	21 (12%)
TSH (µU/ml; normal range: 0.3–4.0 µU/ml)	1.5 (1.0–2.1)
T4 (nmol/l; normal range: 58–140 nmol/l)	90 (77–104)
fT4 (pmol/l; normal range: 11.5–22.5 pmol/l)	16.5 (14.9–18.3)
T3 (nmol/l; normal range: 0.9–2.8 nmol/l)	1.8 (1.5–2.0)
fT3 (pmol/l; normal range: 3.5–6.5 pmol/l)	4.5 (4.1–4.9)
rT3 (nmol/l; normal range: 0.14–0.54 nmol/l)	0.34 (0.28–0.39)
rT3/T3	0.18 (0.15–0.24)
rT3/T4	0.0037 (0.0031–0.0045)
TBG (mg/l; normal range: 10–35 mg/l)	18 (16–21)

Values are given as mean ± SD or median (25th–75th percentiles)

*BMI* body mass index, *CRP* C-reactive protein, *eGFR* estimated glomerular filtration rate, *CKD* chronic kidney disease, *ACR* albumin/creatinine ratio, *fT3* free trioodothyronine, *fT4* free thyroxine, *n.s.* not significant, *rT3* reverse triiodothyronine, *T3* triiodothyronine, *T4* thyroxine, *TBG* thyroxine-binding globulin, *TSH* thyroid-stimulating hormone

### Correlation analysis

**Fig. 1:**
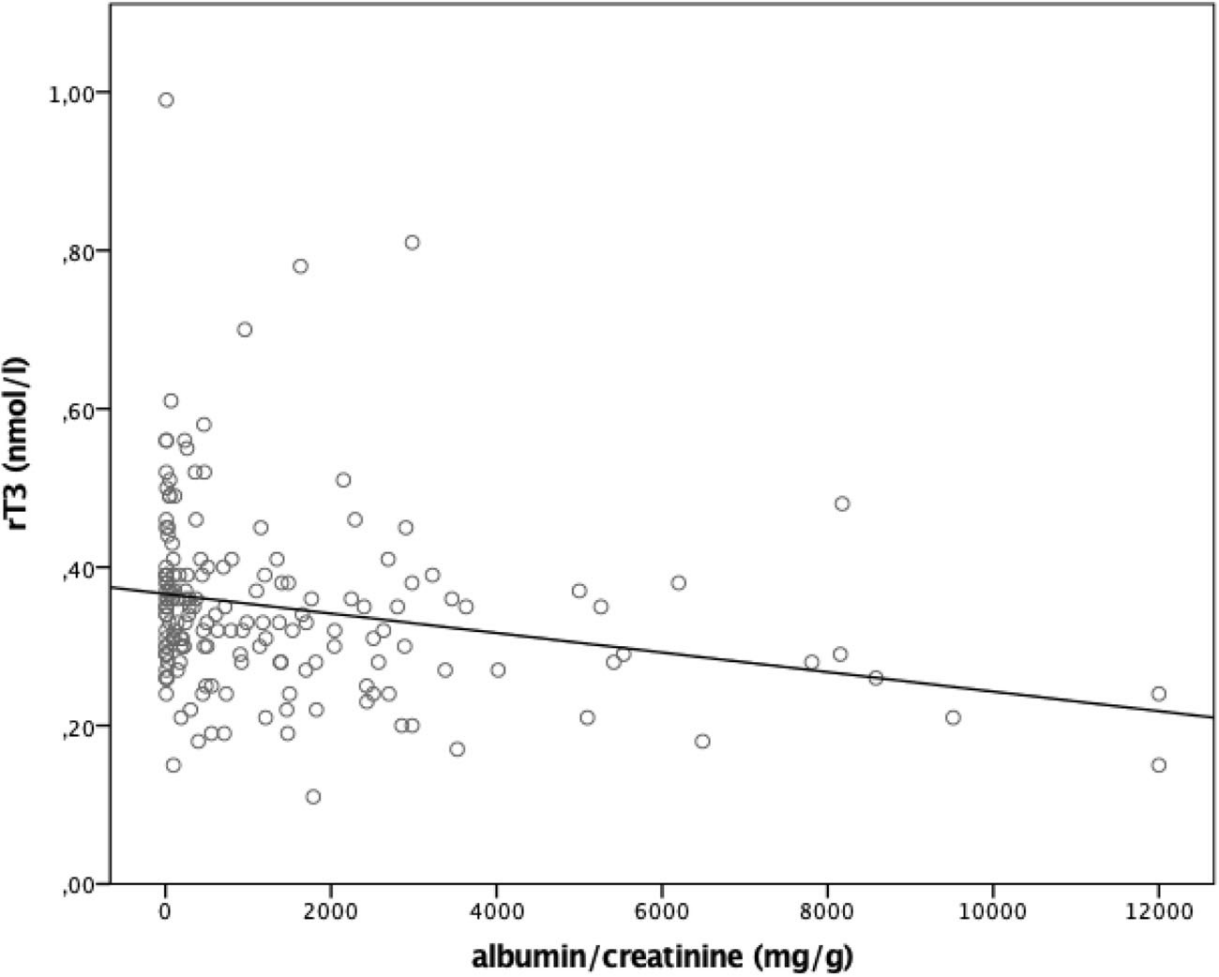
Correlation between rT3 and ACR calculated as Spearman’s rho

We found age to be significantly associated with poor kidney function and damage (eGFR: *r* = −0.403, *p* < 0.0001; ACR: *r* = −0.226, *p* < 0.01). eGFR correlated significantly with peripheral active thyroid hormones, with no influence on TSH or rT3 (see Table 2). ACR exhibited no significant association with TSH or peripheral active thyroid hormones, but it did show a negative association with rT3 (*r* = −0.286; *p* < 0.001) (see Fig. 1), rT3/T3 (*r* = −0.277; *p* < 0.001), and rT3/T4 (*r* = −0.359; *p* < 0.0001). These findings remained significant after correcting for age and CRP. Neither kidney function nor kidney damage correlated with TBG concentrations.

**Table 2:** Correlations of eGFR, albumin mg/g creatinine ratio, and serum albumin with age, analytes of thyroid function, and CRP

		Age	TSH	T4	fT4	T3	fT3	rT3	rT3/T3	rT3/T4	TBG	CRP
eGFR (ml/min/1.73 m^2^)	rho *p* *n*	−0.403 <0.01 184	−0.07 n.s. 179	0.289 <0.01 179	0.196 <0.01 184	0.408 <0.01 178	0.390 <0.01 184	−0.03 n.s. 179	−0.28 <0.01 178	−0.26 <0.01 179	0.233 <0.01 184	−0.161 <0.05 175
ACR (mg/g creatinine)	rho *p* *n*	−0.226 <0.01 184	0.021 n.s. 173	0.057 n.s. 173	0.130 n.s. 180	0.062 n.s. 172	0.025 n.s. 180	−0.28 <0.01 173	−0.28 <0.01 172	−0.36 <0.01 173	0.008 n.s. 177	0.095 n.s. 168
Serum albumin (mg/l)	rho *p* *n*	−0.167 <0.05 183	−0.07 n.s. 172	0.183 <0.05 172	0.071 n.s. 179	0.161 <0.05 171	0.255 <0.01 179	0.167 <0.05 172	0.022 n.s. 171	0.032 n.s. 172	0.143 n.s. 177	−0.244 <0.01 173

*CRP* C-reactive protein, *eGFR* estimated glomerular filtration rate, *ACR* albumin/creatinine ratio, *fT3* free trioodothyronine, *fT4* free thyroxine, *n* number of patients, *n.s.* not significant, *p* *p* value, *rho* correlation coefficient (Spearman’s rho), *rT3* reverse triiodothyronine, *T3* triiodothyronine, *T4* thyroxine, *TBG* thyroxine-binding globulin, *TSH* thyroid-stimulating hormone

### Intergroup analysis

Table 3 summarizes our comparisons of median concentrations of thyroid function analytes among the albuminuria severity subgroups. As expected, median serum albumin differed significantly among the three subgroups. No differences among the subgroups were observed in median TSH, fT4, T3, fT3, or TBG concentrations. For median T4 levels, we noted a significant intersubgroup difference: there was, however, no stepwise drop in T4 from the mild-albuminuria subgroup to severe-albuminuria subgroup. Rather, median T4 concentrations rose from 88 nmol/l in the “mild subgroup” to 97 nmol/l in the “moderate subgroup,” falling then to 78 nmol/l in the “severe subgroup” (*p* < 0.01).

**Table 3:** Biochemical variables according to albuminuria severity (reflected by albumin/creatinine ratio) (*n* = 184)

Variable	Mild (ACR1) (*n* = 80) (0–300 mg/g)	Moderate (ACR2) (*n* = 83) (300–3000 mg/g)	Severe (ACR3) (*n* = 21) (>3000 mg/g)	*p* value
Albumin (mg/g creatinine)	36.5 (10.0–140.0), *n* = 80	1210.0 (63.0–2041.0), *n* = 83	5415.0 (3816.0–8163.5), *n* = 21	<0.0001
Serum albumin (mg/dl)	4.3 (4.1–4.5), *n* = 76	4.2 (3.9–4.4), *n* = 80	3.4 (3.0–3.9), *n* = 19	<0.0001
eGFR (ml/min/1.73 m^2^))	26.06 (18.4–39.7), *n* = 79	25.8 (16.08–50.50), *n* = 83	16.94 (13.02–47.30), *n* = 21	n.s.
TSH (µU/ml)	1.6 (0.8–2.3), *n* = 75	1.4 (1.0–2.0), *n* = 78	1.8 (1.1–2.6), *n* = 20	n.s.
T4 (nmol/l)	88 (75–100), *n* = 75	97 (82–104), *n* = 78	79 (73–90), *n* = 20	<0.05
fT4 (pmol/l)	16.2 (14.7–17.7), *n* = 78	16.8 (15.0–18.8), *n* = 81	16.9 (15.5–19.5), *n* = 21	n.s.
T3 (nmol/l)	1.8 (1.5–2.0), *n* = 73	1.8 (1.6–2.1), *n* = 79	1.8 (1.5–2.0), *n* = 20	n.s.
fT3 (pmol/l)	4.6 (4.0–4.9), *n* = 80	4.4 (4.1–4.9), *n* = 79	4.5 (4.3–5.0), *n* = 21	n.s.
rT3 (nmol/l)	0.36 (0.31–0.40), *n* = 75	0.32 (0.25–0.38), *n* = 78	0.28 (0.22–0.36), *n* = 20	<0.001
rT3/T3	0.21 (0.16–0.27), *n* = 73	0.16 (0.14–0.22), *n* = 79	0.16 (0.12–0.20), *n* = 20	<0.001
rT3/T4	0.0040 (0.0035–0.0049), *n* = 75	0.0034 (0.0028–0.0040), *n* = 78	0.0035 (0.0026–0.0039), *n* = 20	<0.0001
TBG (mg/l)	18 (15–21), *n* = 78	18 (16–21), *n* = 79	17 (15–20), *n* = 20	n.s.

Differences between subgroups were calculated via Kruskal–Wallis tests. Values other than *p* values are given as median (25th–75th percentiles)

*ACR* albumin/creatinine ration, *eGFR* estimated glomerular filtration rate, *fT3* free trioodothyronine, *fT4* free thyroxine, *n.s.* not significant, *rT3* reverse triiodothyronine, *T3* triiodothyronine, *T4* thyroxine, *TBG* thyroxine-binding globulin, *TSH* thyroid-stimulating hormone

**Fig. 2:**
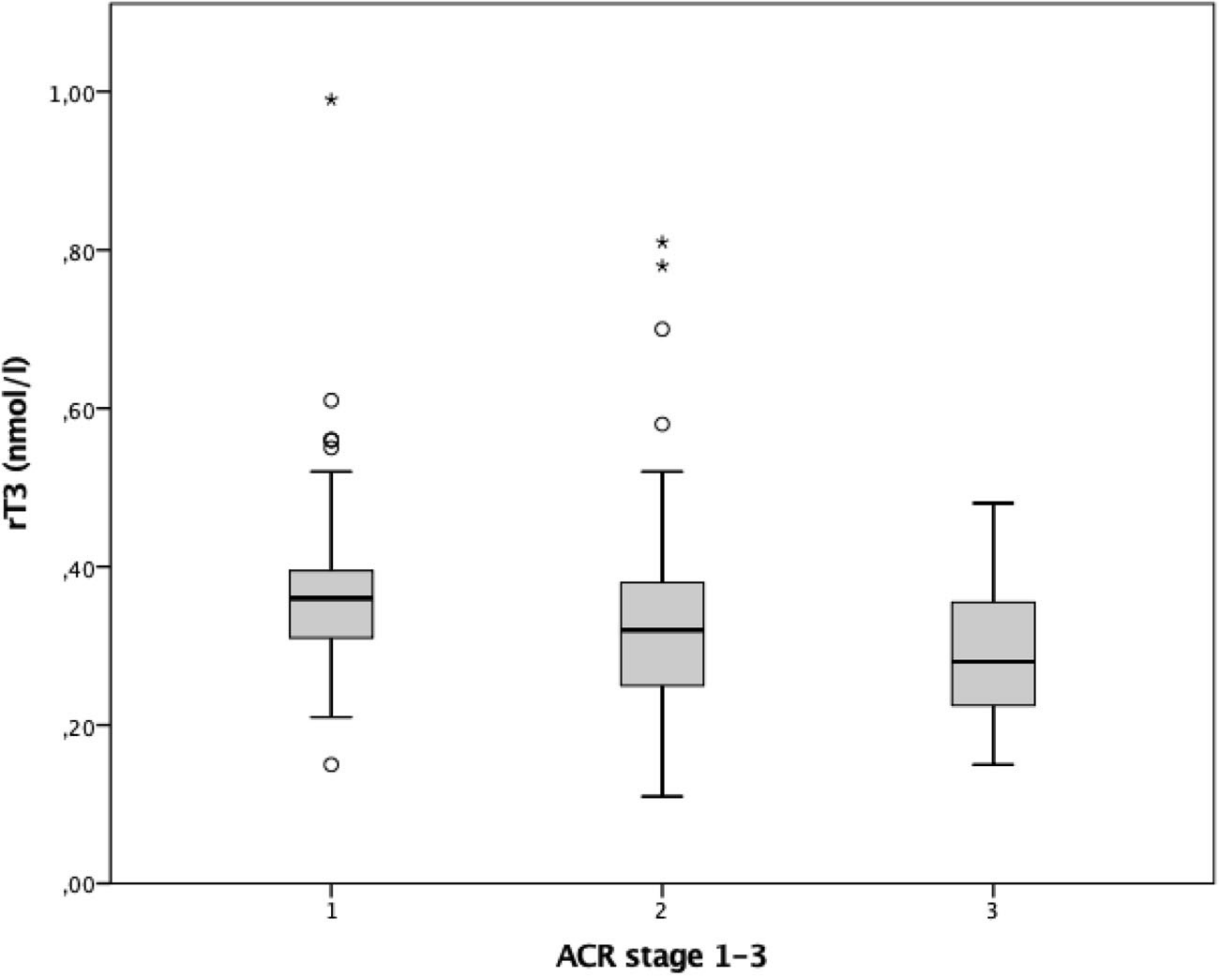
Box plot to show the median rT3 values depending on the ACR stage. The circles (°) correspond to values between 1.5 and 3 interquartile range outside the box. The asterisks (*) correspond to values that are more than three interquartile ranges outside the box

However, the rT3 concentration (ACR1: 0.36 (0.31–0.40) vs. ACR2: 0.32 (0.25–0.38) vs. 0.28 (0.22–0.36), *p* < 0.001) as well as rT3/T4 (ACR1: 0.0040 (0.0035–0.0049) vs. ACR2: 0.0034 (0.0028–0.0040) vs. ACR3: 0.0035 (0.0026–0.0039), <0.0001) and rT3/T3 (ACR1: 0.21 (0.16–0.27) vs. 0.16 (0.14–0.22) vs. 0.16 (0.12–0.20), <0.001) were significantly lower in patients suffering from severe albuminuria than in those whose protein loss was milder. The median quartiles for serum rT3 levels in the three different groups are shown in Fig. 2.

## Discussion

Our study demonstrates that worsening kidney function is associated with falling T4, fT4, T3, and fT3 concentrations, but also that kidney damage even within the nephrotic range had no association with thyroid function in apparently thyroid-healthy patients.

Several investigations have evaluated the relationship between proteinuria and analytes of thyroid function in patients presenting normal kidney function [3–13, 23–26]. In our study, we also included patients who were older (mean age: 63.1 ± 16.9 years; 44.5%, 82/184 age ≥ 65 years) and/or suffering from advanced CKD (stages 4 and 5: 83/184 patients, 45%), with a median eGFR of 25.7 ml/min for the overall study cohort.

We found the relationship between proteinuria and thyroid function analytes to be less pronounced than did many previous working groups. To date, elevated TSH values and the significant loss of TBG and thyroid hormones have been reported in children and adults with proteinuria [3–10, 26, 30]. Sawant et al. detected elevated TSH (5.9 vs. 2.9 mIU/m;) and low T4 and T3 levels in 20 younger patients (ages 12–50 years) with nephrotic syndrome (mean proteinuria: 5.2 ± 1.2 g/day) [31]. Data on kidney function and thyroid antibody status were not provided. Gilles et al. studied 159 TPO antibody-negative patients in early CKD stages (serum creatinine 0.92–1.51 mg/dl) aged a median 52 years and presenting median daily proteinuria of 6.6 g/10 mmol creatinine [23]. Serum albumin was lower in the proteinuria group vs. controls without kidney disease (*n* = 159; 2.9 mg/dl vs. 4.6 mg/dl; *p* < 0.001). They observed a negative correlation between serum albumin and TSH: TSH was slightly higher in their proteinuria group vs. controls (1.84 vs. 1.34 µU/ml), whereas fT4 levels did not differ. Only one patient had overt hypothyroidism.

Yang et al. investigated retrospectively the thyroid hormone status in 211 patients with mean albuminuria of 2.1 ± 2.0 g/day, and found a negative correlation between fT4 and albumin excretion, whereas TSH remained unaffected [27]. However, data on TBG and rT3 concentrations and thyroid antibody status were not reported. Recently, Jain et al. reported significantly higher TSH values in 25 young TPO Ab positive vs. 35 TPO Ab negative patients with mildly impaired renal function and nephrotic syndrome [25]. Li et al. investigated 317 young patients presenting various forms of histologically proven glomerulonephritis with good kidney function, all with proteinuria > 3.5 g/day). They identified 82% with thyroid dysfunction and among them, 55% with (subclinical) hypothyroidism and 45% NTI. Proteinuria was significantly higher in patients with thyroid dysfunction (8.27 g vs. 4.03 g/day). The TPO antibody status was not reported [26].

In contrast to these studies were the results from a Chinese group who evaluated 581 patients with albuminuria [24]. Their patients were divided in subgroups with <30 mg/g creatinine (*n* = 269), 30–300 mg/g creatinine (*n* = 196), and >300 mg/g creatinine (*n* = 116) of albuminuria. Surprisingly, they identified a positive correlation between albuminuria and higher serum T4 and fT4 values in their >300 mg subgroup. However, their >300 mg subgroup’s mean albuminuria only measured 996 ± 843 mg/g creatinine. TSH, T3, and fT3 did not differ among the albuminuria subgroups. They provided no data on either the number of patients within the genuine nephrotic range (>3 g/day) or TPO antibody status. The authors explained the discrepancy between their results and those of other studies partly by racial differences.

We found no association between (subclinical) hypothyroidism and the degree of proteinuria.

Neither TSH, T3, fT3, and T4 nor fT4 differed between subgroups with median albumin/creatinine ratios of 5415 mg/g vs. 36 mg/g. In our study, TBG did not differ among the three albuminuria subgroups or correlate with albumin/creatinine ratio, nor could we detect any correlation between albuminuria and serum TSH, T4, fT4, T3, fT3, or TBG concentrations.

The discrepancies between ours and other studies’ findings might be partly attributable to the fact that our cohort included only thyroid antibody-negative patients. Moreover, as noted above, our patients were older and suffering from more advanced CKD stages than were other cohorts. However, although albuminuria was independent of kidney function in our study, a negative correlation with age was observed. We did not measure T4 or TBG in the urine, so we cannot rule out significant urine loss of these parameters via the kidney, but obviously, our thyroid antibody-negative patients are maintaining a capable, normal thyroid status. Thus, we can speculate that older patients maintain normal thyroid status because their protein loss and presumed T4/TBG loss are less pronounced in their age group.

We detected the most obvious effect of albuminuria severity on rT3 concentrations, namely a significant negative correlation between the serum rT3 concentration and degree of albuminuria. Patients presenting the highest albuminuria revealed lower rT3 values. This relationship remained significant after correcting for age and CRP. Moreover, the lower rT3 values in patients with pronounced proteinuria were independent of kidney function. Our results concur with those of Adlkover et al., who also reported a negative association between proteinuria and rT3 in 28 patients with proteinuria accompanied by normal thyroid hormone parameters [9]. rT3 concentrations in those patients presenting the highest protein loss were even frequently below the lower limit of detectability. Two other investigators reported normal rT3 levels in patients with nephrotic syndrome: Gavin et al. reported normal rT3 levels in ten patients (mean age: 53 years) with a mean GFR of 45 ml/min and with mean daily proteinuria of 11.1 g in the presence of normal T4 concentrations [10]. Feinstein et al. [19] also detected normal rT3 levels in the presence of lower serum T4 and T3 concentrations in 15 nephrotic patients with normal kidney function. Also in Li et al.’s study rT3 did not differ between the 8 and 4 mg proteinuria group [26]. The reason might be that in the presence of the severe glomerulonephritis, those patients have a low T3 and/or low T3/T4 syndrome and therefore rT3 also begins to rise as in the classical form of NTI.

One possible explanation for the rT3 characteristic in patients with protein loss is that in the presence of albuminuria, there is significant rT3 loss in the urine, but in that case, one would also expect serum T3, T4, and TBG to be lower in the severe-albuminuria group. Another explanation comes from Kaptein et al. following their serum rT3 kinetic studies in patients with protein loss [15, 32]: they found low rT3 levels in ten patients with a mean proteinuria of 7 g/day, where they observed impaired binding of rT3 to its binding proteins [32]. Moreover, in the presence of reduced rT3 levels, they identified normal fT4 concentrations levels also suggesting impaired conversion from T4 to rT3 [32].

We noted in addition, as in other forms of NTI, the well-known effects of falling T4, fT4,T3, and fT3 concentrations, all of which correlated positively with eGFR, but stable TSH concentrations appeared also in conjunction with worsening kidney function [14–18]. However, rT3 concentrations did not correlate with eGFR. There is evidence that rT3 is also low or normal even in patients suffering from advanced kidney failure irrespective of proteinuria [15–18].

### Summary

Our results show that in thyroid antibody-negative adults with CKD—even advanced CKD, i.e., stages 4 and 5—albuminuria even in the nephrotic range (>3 mg/g creatinine) reveals no significant association with serum T4, fT4, T3, fT3, TSH, or TBG concentrations. However, the severity of albuminuria, which was independent of renal function, correlated negatively with rT3, and with significantly lower serum rT3 values in patients with albuminuria in the nephrotic range.

CKD patients demonstrate low thyroid hormone values, but in contrast to patients with other forms of NTI, exhibit normal or even low rT3 levels.

Thus, albuminuria might be exacerbating the relatively (low) normal rT3 concentrations in patients with CKD. Nevertheless, clinicians should be aware of (subclinical) hypothyroidism in children and in patients diagnosed with underlying Hashimoto thyroiditis and coexisting nephrotic syndrome.

## Data Availability

Data and material are available on request from the correspondence author.

## References

[1] Ritz E, Orth SR (1999). Nephropathy in patients with type 2 diabetes mellitus. N. Engl. J. Med..

[2] Levey AS, de Jong PE, Coresh J (2011). The definition, classification, and prognosis of chronic kidney disease: a KDIGO Controversies Conference report. Kidney Int..

[3] Burke CW, Shakespear RA (1976). Triiodothyronine and thyroxine in urine. II. Renal handling, and effect of urinary protein. J. Clin. Endocrinol. Metab..

[4] Etling N, Fouque F (1982). Effect of prednisone on serum and urinary thyroid hormone levels in children during the nephrotic syndrome. Helv. Paediatr. Acta.

[5] Ito S, Kano K, Ando T (1994). Thyroid function in children with nephrotic syndrome. Pediatr. Nephrol..

[6] Fonseca V, Thomas M, Katrak A (1991). Can urinary thyroid hormone loss cause hypothyroidism?. Lancet.

[7] Chandurkar V, Shik J, Randell E (2008). Exacerbation of underlying hypothyroidism caused by proteinuria and induction of urinary thyroxine loss: case report and subsequent investigation. Endocr. Pract..

[8] Afrasiabi MA, Vaziri ND, Gwinup G (1979). Thyroid function studies in the nephrotic syndrome. Ann. Intern. Med..

[9] Adlkofer F, Hain H, Meinhold H (1983). Thyroid function in patients with proteinuria and normal or increased serum creatinine concentration. Acta Endocrinol..

[10] Gavin LA, McMahon FA, Castle JN (1978). Alterations in serum thyroid hormones and thyroxine-binding globulin in patients with nephrosis. J. Clin. Endocrinol. Metab..

[11] Benvenga S, Vita R, Di Bari F (2015). Do not forget nephrotic syndrome as a cause of increased requirement of levothyroxine replacement therapy. Eur. Thyroid J..

[12] Junglee NA, Scanlon MF, Rees DA (2006). Increasing thyroxine requirements in primary hypothyroidism: don’t forget the urinalysis!. J. Postgrad. Med..

[13] Halma C (2009). Thyroid function in patients with proteinuria. Neth. J. Med..

[14] Farwell AP (2013). Nonthyroidal illness syndrome. Curr. Opin. Endocrinol. Diabetes Obes..

[15] Kaptein EM, Feinstein EI, Nicoloff JT (1983). Serum reverse triiodothyronine and thyroxine kinetics in patients with chronic renal failure. J. Clin. Endocrinol. Metab..

[16] Witzke O, Wiemann J, Patschan D (2007). Differential T4 degradation pathways in young patients with preterminal and terminal renal failure. Horm. Metab. Res..

[17] Kosowicz J, Malczewska B, Czekalski S (1980). Serum reverse triiodothyronine (3,3’,5’-L-triiodothyronine) in chronic renal failure. Nephron.

[18] De Marchi S, Cecchin E, Villalta D (1987). Serum reverse T3 assay for predicting glucose intolerance in uremic patients on dialysis therapy. Clin. Nephrol..

[19] Feinstein EI, Kaptein EM, Nicoloff JT (1982). Thyroid function in patients with nephrotic syndrome and normal renal function. Am. J. Nephrol..

[20] Meuwese CL, Dekker FW, Lindholm B (2012). Baseline levels and trimestral variation of triiodthyronine and thyroxine and their association with mortality in maintenance hemodialysis patients. Clin. J. Am. Soc. Nephrol..

[21] Rhee CM, Kalantar-Zadeh K, Streja E (2015). The relationship between thyroid function and estimated glomerular filtration rate in patients with chronic kidney disease. Nephrol. Dial. Transplant..

[22] Reinhardt W, Misch C, Jockenhovel F (1997). Triiodothyronine (T3) reflects renal graft function after renal transplantation. Clin. Endocrinol..

[23] Gilles R, den Heijer M, Ross AH (2008). Thyroid function in patients with proteinuria. Neth. J. Med..

[24] Du X, Pan B, Li W (2017). Albuminuria is an independent risk factor of T4 elevation in chronic kidney disease. Sci. Rep..

[25] Jain D, Aggarwal HK, Pavan Kumar YM (2019). Evaluation of thyroid dysfunction in patients with nephrotic syndrome. Med. Pharm. Rep..

[26] Li L-Z, Hu Y, Ai S-L (2019). The relationship between thyroid dysfunction and nephrotic syndrome: a clinicopathological stduy. Sci. Rep..

[27] Yang JW, Han ST, Song SH (2012). Serum T3 level can predict cardiovascular events and all-cause mortality rates in CKD patients with proteinuria. Ren. Fail..

[28] Reinhardt W, Dolff S, Benson S, Broecker-Preuß M, Behrendt S, Hög A, Führer D, Schomburg L, Köhrle J (2015). Chronic Kidney disease distinctly affects relationship between selenoprotein P status and serum thyroid hormone parameters. Thyroid.

[29] Levey AS, Inker LA, Coresh J (2014). GFR estimation: from physiology to public health. Am. J. Kidney Dis..

[30] Dagan A, Cleper R, Krause I (2012). Hypothyroidism in children with steroid-resistant nephrotic syndrome. Nephrol. Dial. Transplant..

[31] Sawant SU, Chandran S, Almeida AF (2011). Correlation between oxidative stress and thyroid function in patients with nephrotic syndrome. Int. J. Nephrol..

[32] Kaptein EM, Hoopes MT, Parise M (1991). rT3 metabolism in patients with nephrotic syndrome and normal GFR compared with normal subjects. Am. J. Physiol..

